# 13 Evaluation of Project „Polygon For Physical Activity of School-Aged Children “for Strengthening Physical Education in Primary Schools without Sport Halls

**DOI:** 10.1093/eurpub/ckae114.167

**Published:** 2024-09-26

**Authors:** Slaven Krtalić, Sanja Music Milanovic

**Affiliations:** Croatian Institute Of Public Health, Zagreb, Croatia; Croatian Institute Of Public Health, Zagreb, Croatia; University of Zagreb, School of Medicine, Andrija Štampar School of Public Health, Zagreb, Croatia

## Abstract

**Purpose:**

In school year 2014/2015, 120 out of 860 central primary schools failed to meet the minimum technical, material and spatial requirements (no access to a sport hall) and, could not carry out the full-time compulsory Physical Education class. To ensure equal opportunities for all pupils to regularly attend Physical Education and help teachers teach to lower grades, the Croatian Institute of Public Health has designed a Set of kinesiological aids and implemented the project “Polygon for the Physical Activity of School-Aged Children”.

**Methods:**

The evaluation of the project was implemented by method of questionnaire. Data were collected in three online questionnaires (Evaluation of the “Polygon” questionnaire 1 (Q1), Evaluation of the “Polygon” questionnaire 2 (Q2), Evaluation of the “Polygon” questionnaire 3 (Q3)) in 120 schools lacking a sport hall. The questionnaires targeted all members of school teams included in the project (two class teachers and one kinesiologist), making up 360 teachers. Online questionnaires were distributed by e-mail to members of school teams for completion, with instructions how to log in, fill it out, and in what deadline. Questions offered multiple answers to choose one or more from, and provided space for additional replies. Respondent teachers voluntarily, completed Q1 at the beginning of second semester, and Q2 and Q3 at the end of the second semester of school year 2015/2016. Response rate for Q1 was 310, for Q2, 178, and for Q3, 121 of overall 360 teachers.

**Results:**

Project evaluation has shown improved conditions and increased pupil interest in Physical Education. A statistically significant difference (χ2=65.30, df = 4, p < 0.001) was noted in teachers perception concerning the suitability of equipment for the youngest pupils before and after the project. The Set was regularly used by 85.59% teachers, whereof 82.88% believed that the pupils loved it. Most teachers believe that this project could help advance the quality of the Physical Education class, and would recommend this Set.

**Conclusion:**

This project presents a positive innovation for the teaching process and a strong potential for lessening the spatial-material problems many schools are experiencing.

**Funding:**

The Croatian Ministry of Health funded the Sets of kinesiological aids.

